# Case Report: Integrated echocardiographic assessment guided Liwen procedure for treating obstructive hypertrophic cardiomyopathy with ventricular aneurysm

**DOI:** 10.3389/fcvm.2023.1278457

**Published:** 2023-11-01

**Authors:** Rui Zhang, Fan Zhao, Jing Wang, Yahong Qin, Tingting Wang, Ai-Ai Chu

**Affiliations:** Department of Echocardiography, First Clinical Medicine School, Gansu Province Hospital, Gansu University of Chinese Medicine, Lanzhou, China

**Keywords:** hypertrophic obstructive cardiomyopathy, hypertrophic cardiomyopathy, transthoracic echocardiography, contrast-enhanced echocardiography, speckle tracking echocardiography

## Abstract

Hypertrophic cardiomyopathy (HCM) is a genetic myocardial disease, with an estimated incidence of 0.2%–6%, and is the main cause of sudden cardiac death (SCD) in young athletes. Left ventricular apical aneurysm (LVAA) is a rare subtype of HCM, accounting for about 5% of HCM patients, and has a higher incidence of cardiovascular adverse events. In cases of hypertrophic obstructive cardiomyopathy with LVAA (HOCM-LVAA) that do not respond adequately to optimized medical therapy, the echocardiography-guided percutaneous intra-myocardial septal radiofrequency ablation (PIMSRA, Liwen procedure) emerges as a promising and effective novel therapeutic approach. In this case report, we present for the first time a comprehensive application of echocardiographic techniques, including TTE, 2-D STE, and contrast enhancement, in the diagnosis, treatment, surgical guidance, and assessment of therapeutic outcomes in a case of HOCM-LVAA.

## Introduction

Hypertrophic cardiomyopathy (HCM) is a genetic myocardial disease, with an estimated incidence of 0.2%–0.6%, and is the main cause of sudden cardiac death (SCD) in young athletes (1–3). Left ventricular apical aneurysm (LVAA) is a rare subtype of HCM, accounting for about 5% of HCM patients, and has a higher incidence of cardiovascular adverse events (4, 5). In cases of hypertrophic obstructive cardiomyopathy with LVAA (HOCM-LVAA) that do not respond adequately to optimized medical therapy, the echocardiography-guided percutaneous intra-myocardial septal radiofrequency ablation (PIMSRA, Liwen procedure) emerges as a promising and effective novel therapeutic approach (6, 7). During the procedure, various echocardiographic images play essential roles in diagnosis, treatment, and outcome evaluation. Transthoracic echocardiography (TTE) enables real-time visualization of cardiac structures, furnishing valuable insights into cardiac chamber dimensions and guiding the procedure (6). Contrast-enhanced echocardiography enables precise assessment structural changes within the left ventricle, crucial for the diagnosis of HOCM-LVAA (7). Strain imaging, 2-D speckle tracking echocardiography (2-D STE) offers valuable insights into alterations in myocardial contractility following the procedure, thereby evaluating the effects of Liwen procedure on HOCM. In this case report, we present for the first time a comprehensive application of echocardiographic techniques, including TTE, 2-D STE, and contrast enhancement, in the diagnosis, treatment, surgical guidance, and assessment of therapeutic outcomes in a case of HOCM-LVAA.

**Figure 1:**
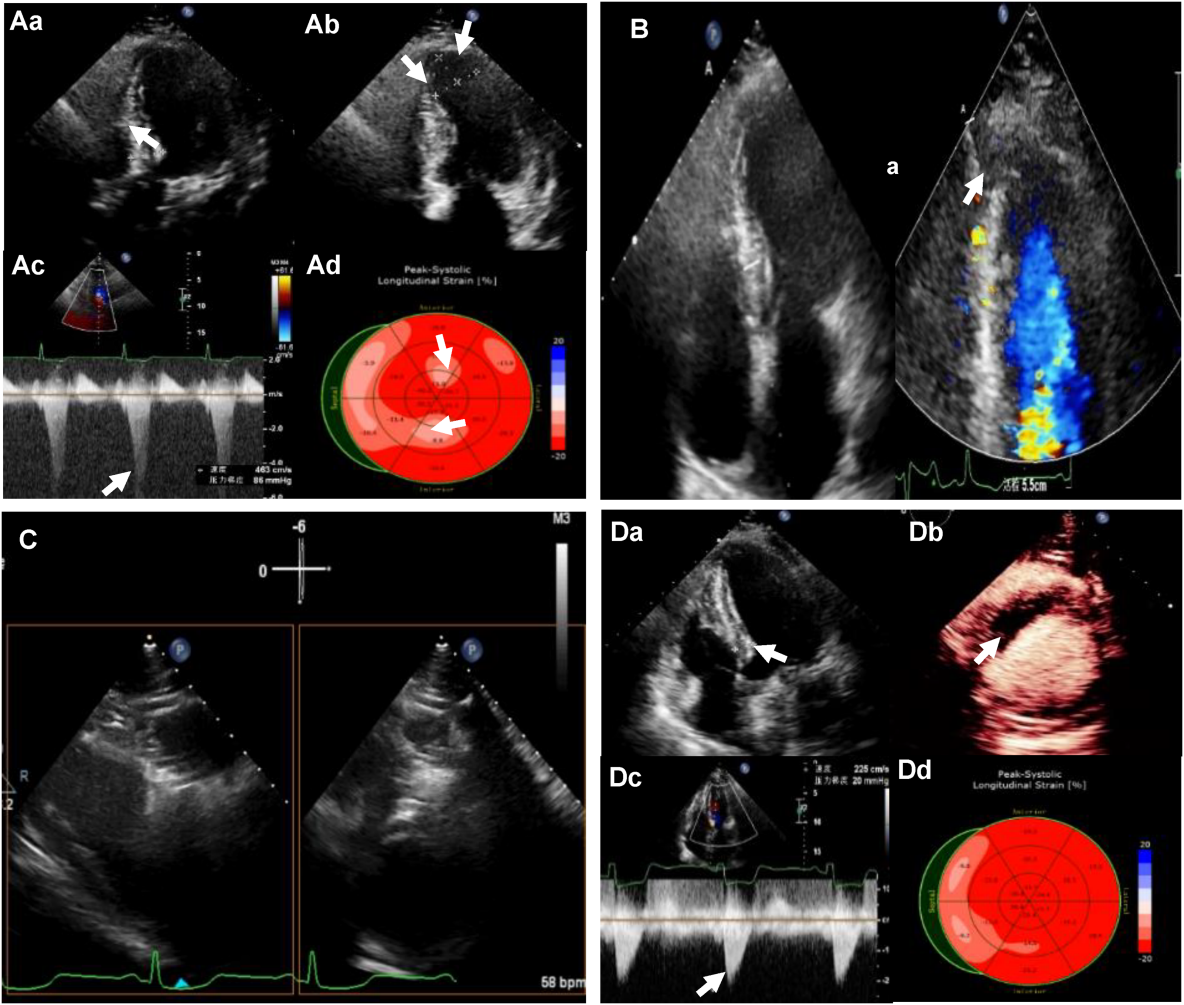
(**Aa**) Preoperative transthoracic echocardiography (TTE): left ventricular (LV) septal thickness of 20mm; (**Ab**) the extent of ventricular aneurysm; (**Ac**) left ventricular outflow tract (LVOT) obstruction at rest; (**Ad**) reduced strain value of local LV apex by strain echocardiography (SE). (**B**) LV apical entrance of the ablation needle guided by TTE. (**C**) Bi-plane showing ablation needle position. (**Da**) LV septal thickness of 12 mm after ablation; (**Db**) myocardial contrast-enhanced echocardiography (MCE) showing a contrast filling defect in the ablation zone; (**Dc**) LVOT gradient of 20 mm Hg after the ablation; (**Dd**) LV apical systolic synchrony detected by SE.

**Figure 2:**
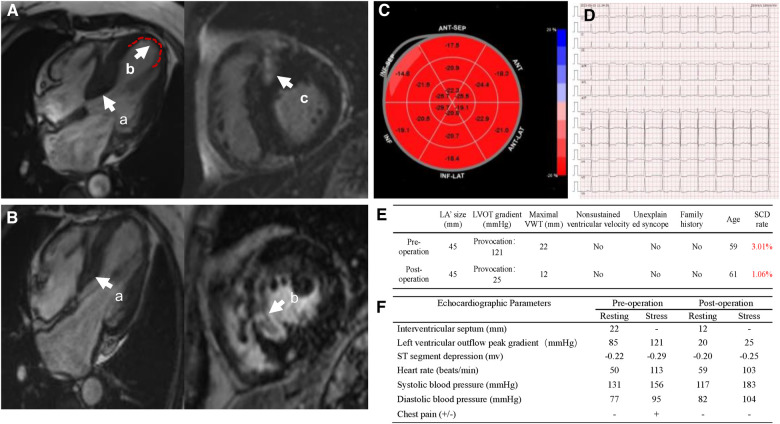
(**A**) Preoperational cardiac magnetic resonance imaging (MRI). (**a**) Thickened interventricular septum, (**b**) the extent of ventricular aneurysm, (**c**) myocardial interstitial fibrosis; (**B**) post-operational cardiac MRI. (**a**) Interventricular septal thickness, (**b**) hypoperfusion area of myocardium at the base of ventricular septum; (**C**) recovered strain values of local LV apex post-operation; (**D**) electrocardiogram post-operation; (**E**) sudden cardiac death score pre- and post-operation; (**F**) stress echocardiographic parameters of pre- and post-operation.

A 57-year-old woman presented with worsen dyspnea after exercise for 5 years. TTE revealed thickening of the interventricular septum (up to 20 mm) and increased flow velocity in the left ventricular outflow tract (LVOT) with a peak velocity of 4.52 m/s and evaluated peak pressure gradient of 85 mmHg. Localized thinning of the ventricular wall at the apex, approximately 5 mm in thickness, was noted, with mild paradoxical motion, covering an area of approximately 19 mm × 30 mm. M-mode echocardiography revealed positive systolic anterior motion of the mitral valve (SAM) and moderate mitral regurgitation. 2-D STE examination were applied for the strain evaluation pre- and post-operation. Pre-operative STE demonstrated the lowest strain value in the basal interventricular septum, decreasing to −5.9%, and the lowest strain value in the left ventricular apex, decreasing to −11.9% (Figure 1A). The patient reported experiencing chest discomfort, shortness of breath, occasional precordial pain, along with fatigue and palpitations, particularly after physical activity. These symptoms would alleviate with rest. Coronary angiography indicated normal origins and courses of the left and right coronary arteries without significant stenosis. Cardiac magnetic resonance imaging (MRI) findings suggested a diagnosis of hypertrophic cardiomyopathy with asymmetrical myocardial hypertrophy, interstitial fibrosis, and left ventricular outflow tract (LVOT) obstruction (Figure 2A). Initial laboratory investigations revealed elevated NT-proBNP levels at 854 pg/ml and cTNT levels at 12.19 ng/L. The electrocardiogram showed sinus rhythm with abnormal Q waves in leads I, II, avL, and V3–V6, as well as horizontal ST-segment depression of 0.05–0.2 mv in leads I, II, III, avF, and V3–V6, accompanied by bidirectional T-wave changes in the same leads. The patient was classified as NYHA III in terms of functional capacity (Figure 2E). Despite undergoing optimal medical therapy, the patient continues to experience symptoms of heart failure, including chest pain, chest tightness, and shortness of breath, classified as NYHA Class III. According to a multidisciplinary consultation, it was recommended that the patient undergo surgical resection and alcohol septal ablation. However, the patient was unwilling to undergo cardiac surgery. Although coronary alcohol septal ablation presents itself as a viable alternative, the ventricular septal branch and location of myocardial hypertrophy is incongruent in this patient. This inconsistency renders it less effective and potentially raises the risk of pacemaker reliance, making it unsuitable for targeted alcohol injection. As a result, we proposed a novel treatment approach in our center, employing radiofrequency ablation for HOCM-LVAA, Liwen procedure.

The patient undergone Liwen procedure under general anesthesia in the supine position. Considering the ventricular aneurysm, we attempted the modified needle technique guided by TTE, in which the approach was reverse insertion to avoid pericardial tamponade caused by insertion into the left ventricle (Figure 1B). The location and characteristics of the aneurysm necessitated a specific approach when choosing the Liwen procedure. Due to the aneurysm's location and features, we opted for a modified insertion approach, transitioning from the conventional B-line approach to the A-line approach. We also rotated the ultrasound probe by 180° and carefully positioned the needle tip along the aneurysmal wall to target the basal interventricular septum for ablation. The presence of the ventricular aneurysm compelled us to pay special attention to the insertion direction and needle tip placement within the aneurysmal myocardium during the Liwen procedure to avoid inadvertent entry into the cardiac chamber and the risk of pericardial tamponade (Figure 1C). The ACT-1530 radiofrequency needle was advanced along the ventricular wall to the hypertrophic portion of the anterior septum under ultrasound guidance. We employed the Philips X5-1 probe with X-plane dual-plane functionality to guide the needle tip's positioning, ensuring it was located in the basal portions of the anterior septum (Zone I, Zone II, Zone III). Radiofrequency energy was then applied, with a maximum power of 80 W, 80 W, and 105 W for each respective zone, and treatment durations of 7 min, 8 min, and 3 min. After ablation, myocardial contrast echocardiography revealed a contrast filling defect in the ablation zone of the interventricular septum. One week later, the thickness of the myocardium in the interventricular septum decreased from 20 mm to 12 mm, and LVOT obstruction was significantly relieved (peak gradient of 20 mmHg). STE showed LV systolic synchrony was achieved, and the apical aneurysm returned to normal appearance of morphology (Figure 1D). One year post the procedure, STE showed an increase in strain values in the basal interventricular septum, reaching −17.5%, and a significant recovery in strain value in the left ventricular apex, returning −22.3%” (with the anterior wall as a reference) (Figure 2B). Through the stress echocardiographic examination, we detected an increased LVOT with a peak velocity of 5.5 m/s and a peak pressure gradient of 121 mmHg in this case. However, 1 year after the procedure, stress echocardiography showed a reduced flow velocity in the LVOT, with a peak velocity of 2.5 m/s and a peak pressure gradient of 25 mmHg (Figure 2F). One-week postoperative assessment, laboratory results indicated NT-proBNP levels at 907 pg/ml and cTNT levels at 5,335 ng/L. Subsequent follow-up examinations at 1 year and 2 years post-procedure showed a reassuring trend with NT-proBNP levels at 801 pg/ml and 657 pg/ml, respectively, and cTNT levels at 13.7 ng/L and 14.8 ng/L, respectively. Postoperative electrocardiography revealed sinus rhythm with horizontal ST-segment depression of 0.05–0.20 mv in leads I, II, III, avF, and V4–V6, with a P-wave duration of 122 ms. Follow-up electrocardiograms at 1 year and 2 years post-procedure continued to show sinus rhythm with horizontal ST-segment depression of 0.05–0.15 mv in leads I, II, avF, and V3–V6, with a P-wave duration of 122 ms (Figure 2C).

## Discussion

We successfully treated a patient with HOCM-LVAA through PIMSRA, Liwen's surgery under various echocardiographic guidance, demonstrating gradient descent, functional recovery, and no post-operative complications. The pathogenesis of HOCM-LVAA includes chronic overload and high pressure in the apex of the heart, ventricular remodeling, and gradual exacerbation of myocardial hypertrophy, leading to local damage and dilation of the ventricle, and irreversible changes in the structure and function of the ventricle, eventually resulting in the formation of a ventricular aneurysm (8). Liwen's Procedure is a novel interventional surgery that uses percutaneous access to puncture the ventricular wall and deliver a certain amount of radiofrequency energy to ablate the obstructive myocardial tissue, thus reducing outflow tract obstruction and improving heart function (7, 9). The indications for Liwen procedure primarily include patients who, despite receiving maximum tolerated doses of optimized medication, continue to experience severe symptoms such as breathlessness, chest pain, or exercise-induced syncope, with their cardiac function classified as NYHA Grade III/IV or CCS Grade III/IV. After a comprehensive evaluation of the patient's clinical condition, which suggested a low risk of arrhythmias and a lower likelihood of sudden cardiac death (SCD), implantable cardioverter-defibrillator (ICD) implantation was not deemed necessary as a part of the overall therapeutic approach in this case. In addition, Liwen procedure indications also include a left ventricular outflow tract gradient (LVOTG) or left ventricular intracavitary pressure difference of ≥50 mmHg at rest and during provocation, as determined through echocardiographic assessment. Patients who do not meet the above criteria but exhibit other high-risk factors or severe symptoms may also be considered for Liwen surgery. In comparison, alcohol septal ablation (ASA) has more stringent indications. ASA is recommended for patients who meet criteria including clinical, hemodynamic, and morphological indications (10). Clinical indications involve persistent symptoms despite standard medication treatment, a baseline heart rate around 60 beats per minute, and classification as NYHA Grade III/IV or CCS Grade III for chest pain. High-risk factors or exercise-induced syncope may also qualify. Hemodynamic indication requires specific LVOTG values at rest or after provocation. Morphological indications encompass septal thickness, obstruction location, and various anatomical considerations, including the absence of papillary muscle involvement. Coronary angiography and myocardial acoustic imaging are used to identify suitable septal branches for ablation. Therefore, both Liwen surgery and alcohol septal ablation are viable treatment options for HOCM patients with ventricular septal hypertrophy. However, Liwen surgery is generally associated with less trauma and offers greater precision in interventional treatment. Guided by echocardiography, Liwen surgery enables physicians to control the treatment area more accurately, reducing the associated risks. Different areas can be treated during Liwen procedure as needed to alleviate myocardial hypertrophy and achieve better outcomes. Alcohol septal ablation, on the other hand, uses coronary artery catheterization techniques, which, while more convenient in some situations, can lead to discomfort due to the injected alcohol causing coronary artery spasm or damage, potentially resulting in coronary artery stenosis or other complications (11–13). For HOCM patients with ventricular septal hypertrophy, Liwen surgery may be an effective treatment option, while alcohol septal ablation may be more suitable for specific patients, especially those who are not suitable for Liwen surgery or require rapid symptom relief. Wang et al. reported 68 patients with drug resistant HOCM who underwent PIMSRA with Liwen procedure (14). All procedures were technically successful, and the ablation functioned without errors. The research demonstrates that the Liwen procedure effectively reduces LVOT gradients over a 12-month follow-up period, indicating its potential as an alternative treatment for HOCM patients with promising safety and efficacy (14).

Although electrocardiography, cardiac magnetic resonance imaging, and cardiac catheterization serve as auxiliary diagnostic methods for HOCM-LVAA, the primary diagnosis counts on echocardiography. TTE can be used to evaluate myocardium hypertrophy and the morphological characteristics of the ventricular aneurysm, enabling accurate early diagnosis. In this case, we utilized TTE and left ventricular opacification (LVO) to provide high-resolution cardiac images, clearly showing the structure and thickness of the ventricular myocardium, thus confirming the diagnosis of HOCM-LVAA. TTE accurately evaluates the morphological features, location, and size of the apical aneurysm, which provides essential information for surgical intervention. Due to the aneurysm's location and features, we modified the insertion approach, transitioning from the conventional B-line to the A-line. We also rotated the ultrasound probe by 180° and precisely positioned the needle tip along the aneurysmal wall to target the basal interventricular septum for ablation, guided by echocardiography. This adjustment was crucial in preventing inadvertent entry into the cardiac chamber and minimize the risk of pericardial tamponade.

For patients with HOCM-LVAA, early intervention is especially crucial. The treatments include medical therapy, surgical septal myectomy, and percutaneous septal ablation to alleviate left ventricular outflow tract obstruction, improve heart function, relieve symptoms, and prevent adverse cardiovascular events. Considering the specific conditions of patients with ventricular aneurysms, individualized treatment is required, considering factors such as age, severity of the condition, and coexisting complications. STE is an emerging ultrasound technique that measures myocardial strain, reflecting myocardial contractile function. In this case, we used strain echocardiography to not only detect reduced strain in the basal segment of the septum but also to identify decreased strain values in the left ventricular apical myocardium, indicating the presence of myocardial dysfunction in the apical region. As the patient still experienced symptoms of heart failure despite optimized medical therapy, we suggested this patient undergo percutaneous septal ablation, Liwen's Procedure. In this case, the patient was diagnosed with hypertrophic cardiomyopathy, which met the indications for Liwen's Procedure. During surgery, we adopted an improved needle technique guided by TTE, accurately locating the ventricular aneurysm, and determining the position and depth of radiofrequency energy delivery to maximize the relief of outflow tract obstruction. Immediate myocardial opacification after surgery revealed a local filling defect in the region of septal ablation, providing an accurate assessment of the surgical outcome. One-week post-operation left ventricular opacification showed that the myocardial thickness of the septum decreased from 20 mm to 12 mm, with a significant alleviation of LVOT obstruction (peak gradient of 20 mmHg), indicating the disappearance of the ventricular aneurysm and restoration of a normal appearance. STE showed synchronous contraction of the left ventricle, suggesting a significant improvement in apical myocardial contractility.

In conclusion, the Liwen Procedure introduces novel avenues for treating HOCM-LVAA, with echocardiographic approaches playing a pivotal role in diagnosis, surgical guidance, and outcome assessment (15). For patients with HOCM-LVAA, Liwen's Procedure alleviates left ventricular outflow tract obstruction and significantly improves cardiac function. The combined application of novel echocardiographic techniques refines disease diagnosis and treatment, enhancing the precision and efficacy of Liwen's Procedure. We propose that the integration of novel echocardiographic approaches to guide Liwen's Procedure not only facilitates the diagnosis and management of HOCM-LVAA but also substantially enhances patients' quality of life and long-term prognosis.

## Data Availability

The raw data supporting the conclusions of this article will be made available by the authors, without undue reservation.
